# Household catastrophic medical expenses in eastern China: determinants and policy implications

**DOI:** 10.1186/1472-6963-13-506

**Published:** 2013-12-05

**Authors:** Xiaohong Li, Jay J Shen, Jun Lu, Ying Wang, Mei Sun, Chengyue Li, Fengshui Chang, Mo Hao

**Affiliations:** 1 Research Institute of Health Development Strategies, School of Public Health, Fudan University, Shanghai, China; 2 Department of Health Care Administration and Policy, University of Nevada, Las Vegas, USA

## Abstract

**Background:**

Much of research on household catastrophic medical expenses in China has focused on less developed areas and little is known about this problem in more developed areas. This study aimed to analyse the incidence and determinants of catastrophic medical expenses in eastern China.

**Methods:**

Data were obtained from a health care utilization and expense survey of 11,577 households conducted in eastern China in 2008. The incidence of household catastrophic medical expenses was calculated using the method introduced by the World Health Organization. A multi-level logistic regression model was used to identify the determinants.

**Results:**

The incidence of household catastrophic medical expenses in eastern China ranged from 9.24% to 24.79%. Incidence of household catastrophic medical expenses was lower if the head of household had a higher level of education, labor insurance coverage, while the incidence was higher if they lived in rural areas, had a family member with chronic diseases, had a child younger than 5 years old, had a person at home who was at least 65 years old, and had a household member who was hospitalized. Moreover, the impact of the economic level on catastrophic medical expenses was non-linear. The poorest group had a lower incidence than that of the second lowest income group and the group with the highest income had a higher incidence than that of the second highest income group. In addition, region was a significant determinant.

**Conclusions:**

Reducing the incidence of household catastrophic medical expenses should be one of the priorities of health policy. It can be achieved by improving residents’ health status to reduce avoidable health services such as hospitalization. It is also important to design more targeted health insurance in order to increase financial support for such vulnerable groups as the poor, chronically ill, children, and senior populations.

## Background

Out-of-pocket payment for medical services is very high in most developing countries and the household’s catastrophic medical expenses are being used as one of the indicators measuring whether out-of-pocket payment disrupts material living standards of individuals and households [1,2]. Reducing incidence of household catastrophic medical expenses is one of the objectives of health policy [1]. Understanding the incidence of catastrophic medical expenses and its determinants is the basis for developing effective health policies to address this problem.

Studies on household catastrophic medical expenses in China have so far focused on relatively less developed regions, central China and western China, especially rural areas in these regions [3]. Most of the studies merely focus on descriptive analysis on the situation and efforts of identifying underlying factors that are associated with catastrophic medical expenses have been lacking [3]. Furthermore, based on our literature review, studies on this problem in eastern China, a relatively developed region, are rare. Sun and colleagues examined the effect of the New Cooperative Medical Scheme on catastrophic medical expenses but their analysis did not consider socioeconomic factors [4].

It has been reported that both affluent and poor residents in eastern China are concerned about the high out-of-pocket payment of medical expenses that can seriously compromise their living standard [5]. However, the incidence and determinants of household catastrophic medical expenses in this region remain unknown. It is important to identify the influencing factors, which helps find the specific approaches to solve the problems. This study estimated the incidence of household catastrophic medical expenses and then identified its associated factors. In addition, comparison between eastern China and other part of China is also conducted.

## Methods

### Study setting

It was a cross-section survey conducted in eastern China. We conducted a health care utilization and expenses survey in three cities, Shanghai, Changzhou in Jiangsu province, and Weifang in Shandong province, in eastern China in 2008. The data are not openly available to the public. Shanghai is a well-developed municipality (equivalent to province) directly under control of the central government. In 2010, The national per capita GDP was 29,992 in the mainland that year [6]. Shanghai was ranked the first in terms of per capita GDP among the 31 provinces or municipalities in the mainland in China, with a GDP per capita of 76,074 yuan [6]. Shanghai city consists of 8 central districts and 9 suburban districts. Because the economic level of the central districts is very high and there are only urban residents in the central districts, they are not ideal representatives for eastern China. Among the 9 suburban districts, we selected Jiading district as the sample area with relatively high economic level among the suburban districts, which could better represent the well-developed areas in eastern China. Jiangsu province and Shandong province were ranked the 4^th^ (52,840yuan) and 9^th^ (41,106 yuan) on per capital GDP, respectively [6]. Changzhou city in Jiangsu province was selected for our survey and its per capita GDP was 67,327 yuan [7]. Weifang city in Shandong province was selected and its per capita GDP was 35,260 yuan in 2010 [8].

### Sampling

After selecting the study areas, Jiading in Shanghai, Changzhou in Jiangsu and Weifang in Shandong, we sampled the residents using a stratified method. In recent years, though town merging has taken place in the three areas, there has not been a significant change in residents’ daily life. Therefore, we still used the previous allocation of towns when sampling. There were a lot of differences in living standard between residents and rural residents, so we selected urban residents and rural residents respectively, namely, we selected urban residents in Jiading, rural residents in Jiading, rural residents in Changzhou, urban residents in Weifang and rural residents in Weifang, respectively.

We take sampling of rural households in Changzhou as an example to demonstrate the sampling design, sample size calculation and response rate.

**Figure 1:**
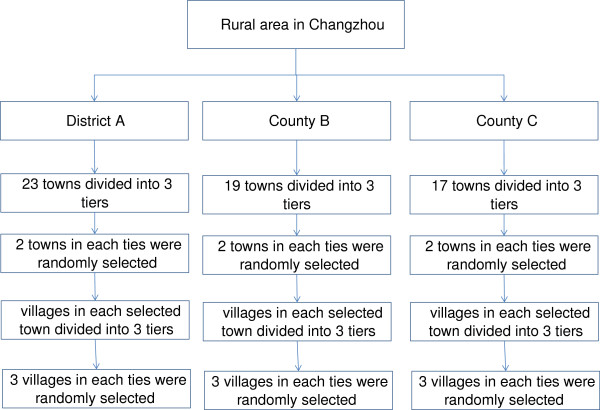
Flow diagram of sampling of rural households in Changzhou.

In 2008, Changzhou consisted of 5 districts and 2 counties, with 1.2221 million households, and 39% of the population was rural resident. Rural residents mostly lived in one district (District A) and the two counties (County B and County C). District A, County B and County C had 23, 19 and 17 towns, respectively. We used the stratified random sampling method. Firstly, all the towns in District A, County B and County C respectively were divided into 3 tiers in respect to their income levels. In each tier, 2 towns were selected randomly. Secondly, in each of the selected towns, all villages were grouped into three tiers: near to, middle-distance from, and far away from the centre point of the town. In each tier, 3 villages were randomly selected. Finally, 40–60 rural households were randomly selected from each of the villages. The sampling was shown in the Figure 1.

We used the simple random sampling formula to calculate the sample size of rural households in Changzhou, because we only had limited literature and more parameters were needed in the stratified sample size calculation formula. After we got the total sample needed, we allocated the sample size in each selected village using probability proportionate to size sampling (PPS) [9].

The simple random size calculation formula [10]:

$$n=\frac{u_{\propto }^{2}\pi \left(1-\pi \right)}{\delta^{2}}$$

Let δ = 0.025, u = 1.96, α = 0.05. The incidence of catastrophic medical expenses in the literature range from 4% to 20% [3,4,11], and we supposed π ranged from 4% to 25%. We got the biggest size of 1152 households. Taking incomplete questionnaires into account, we should survey about 1300 households. The local government wanted to better understand the situation of Changzhou and provided organizational support, and finally we expanded the sample size to 2700 households. Finnaly, according to rural households in Changzhou, 54 villages in 18 towns were selected, and 2703 households were surveyed, and 128 households were excluded due to incomplete data. Therefore the response rate was 95.26%.

Similarly, households in other regions were selected using this stratified method. The sample size of rural households is the smallest compared with other group of households, and reasons are as following. As for rural households in Jiading in Shanghai, the incidence of catastrophic medical expenses was 11.78% in Shanghai based on the literature [11], and we supposed π ranging from 7% to 16%. We got the biggest size of 784 households. Taking incomplete questionnaires, we should survey at about 850 households.

In the 3 regions, a total of 12070 households were interviewed, and the percentage of households in different regions is shown in the Table 1. After excluding 493(4.08%) households that did not have complete information, we finally have 11577 households in our study with an overall response rate of 95.92%.

**Table 1:** Household characteristics and out-of-pocket medical expenses

**Variable**	**Percentage (%)**
Region	
Shanghai	27.62
Changzhou	22.34
Weifang	50.13
Household head’s registration status	
Rural	68.42
Urban	31.58
Household head’s education level	
Elementary school or lower	38.60
Middle school	42.87
High school	14.14
College or higher	5.40
Household head’s health insurance status	
Uninsured	5.75
Labor insurance	12.44
Urban resident insurance	8.76
New cooperative medical scheme (NCMS)	61.93
other social insurance	12.12
Household head’s having commercial insurance	
Yes	7.40
No	92.60
Number of family members with chronic disease	
None	70.61
1	21.21
2	7.48
3 or more	0.70
Number of family members with an age of 65 or older	
None	70.99
1	15.43
2 or more	13.58
Number of family members younger than 5 years old	
None	86.47
1	13.30
2 or more	0.22
Household size	
1	4.88
2	29.95
3	30.09
4 or more	35.08
Household head’s marriage status	
Unmarried	2.86
Married	90.36
Divorced	0.83
Other	5.95
Annual per capita expenses (yuan)	
Shanghai	8656.2
Changzhou	6722.6
Weifang	4505.7
Annual per capita out-of-pocket medical expenses (yuan)	
Shanghai	885.4
Changzhou	632.9
Weifang	516.9
Number of hospitalization in last 12 months	
None	92.12
1	7.13
2	0.58
3 or more	0.16

### Organization of survey

The survey was organized by the local Health Bureau in Jiading, Changzhou and Weifang. Investigators consisted of teachers, graduate students and undergraduate students from Fudan University and Weifang Medical College, and local CDC (Center for Disease control) staff. All the investigators were trained before they went to investigate the residents. The residents were interviewed face-to-face at their home. The survey was conducted in July to October in 2008.

We gained approval (IRB#08–03–0130) for the study from the Medical Research Ethics Committee, School of Public Health, Fudan University (IRB00002408&FWA00002399).

### Measures and questionnaire

We, using a structured questionnaire (see Additional file 1), conducted face-to-face interviews. The questionnaire consisted of three parts: (1) household member’s age, sex, marital status, education level, employment status, health insurance status, and self-reported chronic illness status; (2) the amount of expenses of the household in 2007, covering food, daily necessities, transportation and communication, accommodation, education and entertainment, health care expenses, and other spending, (3) out-of-pocket health care payment including outpatient, hospitalization, and self-care in 2007.

We used annual expenses per capita as an indicator to measure household’s living standard. First of all, we used the information about the amount of expenses of the household in 2007, to calculate annual expenses per capita of the household [2]. Secondly, we divided households into five groups based on quintiles of their annual per capita expenses of the household.

We used a method introduced by another study [12] to calculate the incidence of catastrophic medical expenses based on four cut-off levels—equal to or greater than of 40% of capacity to pay, 30% of capacity to pay, 20% of capacity to pay, 10% of capacity to pay. When we analyzed the determinants of catastrophic medical expenses, we used a high threshold of payments of at least 40% of a household’s capacity of pay. A household’s capacity to pay is defined as total expenditure of the household remaining after basic subsistence needs have been met.

Capacity to pay of the i*th* household is:

$$\text{CTP}=\text{EX}P_{i}-SE_{45-55i}$$

Subsistence expenditure (*SE*_*45-55i*_) is the average food expenditure of households in the 45th to 55th percentile, adjusted for the size of the *i*th household. For households whose total spending is below the estimated subsistence need, capacity to pay is taken as the observed nonfood spending.

### Statistical analysis

The multi-level logistic model, supported by Stata11.0, was used to analyse factors associated with catastrophic medical expenses [13]. Because the households being interviewed were not selected by simple random sampling from the view of all the households, the households in the same region were not independent. Using the multi-level model took into account this potential correlation within group. The dependent variable of the multivariable analysis was a dichotomous variable, indicating whether or not catastrophic medical expenses occurred in a household. If a household’s out-of-pocket health care payment exceeded 40% of the household’s capacity-to-pay [12], it was identified as incurring catastrophic medical expenses. Mainly based on literature review [3,14], we defined the independent variables as shown in Table 2. Independent variables included the level-1 variables (the household level variables) and the level-2 variable (the region level variable). Preliminary analysis showed the Variance Inflation Factor (VIF) values of the independent variables being 2.36, indicating that the multicolinearity issue did not exist [15].

**Table 2:** Dependent variable and independent variables

**Variable**	**Description**
Dependent variable	1 if household incurred catastrophic medical expenses
	0 otherwise
Independent variable	
Household level	
Household head’s residency status	1- rural, 0 - urban
Household head’s ethnicity	1- han, 0 - other
Household head’s education level	
Primary school or lower	Reference
Middle school	1 if yes, 0 otherwise
High school	1 if yes, 0 otherwise
College or higher	1 if yes, 0 otherwise
Household head’s marriage status	
Unmarried	Reference
Married	1 if yes, 0 otherwise
Divorced	1 if yes, 0 otherwise
Other	1 if yes, 0 otherwise
Household head’s social health insurance	
Uninsured	Reference
Labor insurance	1 if yes, 0 otherwise
Urban residents insurance	1 if yes, 0 otherwise
New Cooperative Medical Scheme(NCMS)	1 if yes, 0 otherwise
Other social insurance	1 if yes, 0 otherwise
Household head’s private health insurance	1 if yes, 0 no
Household head’s occupation	
Manager in government, enterprises and institutions	Reference
Private entrepreneurs and managers	1 if yes, 0 otherwise
Professional and technical personnel	1 if yes, 0 otherwise
Ordinary administrative in government, enterprises and institutions	1 if yes, 0 otherwise
Employees in business or service industry	1 if yes, 0 otherwise
Primary businesses	1 if yes, 0 otherwise
Urban worker	1 if yes, 0 otherwise
Farmers engaged in non-agricultural labor	1 if yes, 0 otherwise
Farmer	1 if yes, 0 otherwise
Unemployed	1 if yes, 0 otherwise
Tired	1 if yes, 0 otherwise
Other	1 if yes, 0 otherwise
Household head’s age group	
<25 years	Reference
> = 25 and <30 years	1 if yes, 0 otherwise
> = 30 and <35 years	1 if yes, 0 otherwise
> = 35 and <40 years	1 if yes, 0 otherwise
> = 40 and <45 years	1 if yes, 0 otherwise
> = 45 and <50 years	1 if yes, 0 otherwise
> = 50 and <55 years	1 if yes, 0 otherwise
> = 55 and <60 years	1 if yes, 0 otherwise
> = 60 and <65 years	1 if yes, 0 otherwise
> = 65 and <70 years	1 if yes, 0 otherwise
> = 70 and <75 years	1 if yes, 0 otherwise
> = 75 years	1 if yes, 0 otherwise
Household head’s sex	1- female, 0-male
Number of persons with chronic disease	
None	Reference
1	1 if yes, 0 otherwise
2	1 if yes, 0 otherwise
3 or more	1 if yes, 0 otherwise
Number of persons 65 years old or older	
None	Reference
1	1 if yes, 0 otherwise
2 or more	1 if yes, 0 otherwise
Number of persons younger than 5 years old	
None	Reference
1	1 if yes, 0 otherwise
2	1 if yes, 0 otherwise
Number of hospitalizations in last 12 months	
None	Reference
1	1 if yes, 0 otherwise
2	1 if yes, 0 otherwise
3	1 if yes, 0 otherwise
Income quintile	
1st (top)living standard quintile	Reference
2nd living standard quintile	1 if yes, 0 otherwise
3rd living standard quintile	1 if yes, 0 otherwise
4th living standard quintile	1 if yes, 0 otherwise
5th(bottom)living standard quintile	1 if yes, 0 otherwise
Household size	
1	Reference
2	1 if yes, 0 otherwise
3	1 if yes, 0 otherwise
4 or more	1 if yes, 0 otherwise
Area level	
Shanghai	Reference
Changzhou	1 if yes, 0 otherwise
Weifang	1 if yes, 0 otherwise

In this study, it is more in line with the characteristics of the data to choose the multi-level than the ordinary logistic model. Multi-level model decomposes random error to the corresponding level, and it builds complex error hierarchical structures. Multi-level model has regression equation similar to ordinary regression model, but the residual was decomposed into different hierarchical structures, therefore it can improve the effect of the model fit. In the ordinary regression model, these independent variables are all on the same level, which results in the loss of the information contained in data with hierarchical structures.

The data of this study, it is obviously that the independent variables belong to different levels. For example, the viable “family member older than 65y” is a household-level variable, however, the variable “region” is an area level one, which represents the general situation of the region’s socio-economic status. Due to lack of more detailed data on region, this study uses the integrated variable “region”, which in fact reflects the differences among regions in terms of medical services price, culture background, health insurances and other information. In China, even the same health insurance, such as New Rural Cooperative Medical, the extent of compensation in different regions is usually different [4]. Based on the characteristics of the data of this study, it is more suitable to use the multi-level model which reflects the hierarchy of the data.

## Results

Household characteristics and out-of-pocket medical expenses are displayed in Table 1. Among the total of 11,577 households, 68.42% were located in rural areas, 29.39% had one or more persons with chronic disease(s), 29.01% had one or more persons with an age of 65 or older, and 13.52% had one or two children younger than 5 years old. Further, 7.87% of the households had at least one person being hospitalized in the last 12 months. Annual per capita out-of-pocket medical expenses were 885.4 yuan, 632.9 yuan, and 516.9 yuan, accounting for 10.23%, 9.41% and 11.47% of annual total expenses, in Shanghai, Changzhou, and Weifnag, respectively.

Based on availability of public data, we compared the annual per capita income in this study and public data as shown Table 3, which showed that indicators in this study were similar to the public data except a little difference.

**Table 3:** Comparison of annual per capita income in this sample and public data (yuan)

**Group of households**	**Sample data**	**Public data**
Urban Shanghai	19291	19770*
Rural Shanghai	11502	11416*
Rural Changzhou	10908	10171**
Urban Weifang	12922	13476***
Rural Weifang	6151	6278***

*Public data are from: Report of National Economic and Social Development in 2007 ( http://tjj.jiading.gov.cn/website/pages/content_0.htm?channel=23&id=28).

**Public data from: Report of National Economic and Social Development Indicators in 2007 ( http://www.cztjj.gov.cn/node/tjsj_2/2011-8-18/1856031655.html).

***Public data are from: Shandong Statistical Yearbook in 2007 ( http://www.stats-sd.gov.cn/tjsj/nj2008/indexch.htm).

Table 4 shows the incidence of catastrophic medical expenses based on the four cut-off levels. At the 40% level, 9.24% - 24.79% of the households incurred catastrophic medical expenses that varied across different areas. Urban areas of Shanghai had the lowest incidence whereas rural areas of Shandong had the highest. At the 40% cut-off level, the 4^th^ quintile had the highest incidence of catastrophic medical expenses and the 2^nd^ quintile had the lowest.

**Table 4:** Incidence of catastrophic medical expenses by different cut-offs (%)

	**Out-of-pocket medical expenses as a percentage of total non-food expenses**			
	**> = 40%**	**> = 30%**	**> = 20%**	**> = 10%**
Area				
Shanghai - urban	7.64	12.39	19.37	37.39
Shanghai - rural	11.48	17.55	27.15	47.02
Changzhou - rural	11.61	15.57	23.18	41.75
Weifang - urban	13.64	20.16	29.99	49.34
Weifang - rural	22.95	30.07	40.47	58.42
Income level				
Top quintile	10.84	14.46	18.65	32.9
2^nd^ quintile	9.24	14.43	23.11	40.65
3^rd^ quintile	11.06	17.07	26.66	47.93
4^th^ quintile	24.79	31.49	40.60	60.3
Bottom quintile	21.06	28.53	41.82	61.16
Total	15.40	21.2	30.17	48.59

Table 5 shows the result of multi-level mode. The whole model is significant as shown in the table. The test of rho shows that region was significantly associated with the incidence of catastrophic medical expenses, which means that households within region were correlated. Most of household-level variables were also significant. As compared to the top income group, the 4^th^ and 5^th^ (bottom) income group were more likely to incur catastrophic medical expenses. Households whose heads were rural residents were more likely to incur catastrophic medical expenses. The level of education of household heads was also negatively associated with catastrophic medical expenses. Households who had one, two, three or more than family members with chronic diseases had high incidence of catastrophic medical expenses. Households who had one or two family members with older than 65y had high incidence of catastrophic medical expenses. Households who had one or two family members younger than 5 years old had high incidence of catastrophic medical expenses. As compared with households whose heads were uninsured, households whose heads had labor insurance coverage were less likely to incur catastrophic medical expenses, while households whose heads had urban resident health insurance or new cooperative medical scheme (NCMS) coverage were comparable in regard to incurring catastrophic medical expenses.

**Table 5:** Determinants of incidence of household catastrophic medical expenses

**Variables**	**OR (95%CI)**	**P-Value**
Income level		
2^nd^ quintile vs top quintile	0.89(0.71–1.10)	0.281
3^nd^ quintile vs top quintile	1.17(0.94–1.44)	0.152
4^th^ quintile vs top quintile	3.25(2.68–3.94)	0.000
Poorest quintile vs top quintile	2.34(1.91–2.85)	0.000
Household size		
2 vs 1	0.77(0.55–1.07)	0.117
3 vs 1	0.50(0.35–0.69)	0.000
4 vs 1	0.45(0.32–0.63)	0.000
Household head’s registration status (rural vs urban)	1.47(1.17–1.83)	0.001
Family member with chronic disease		
1 vs 0	2.07(1.80–2.38)	0.000
2 vs 0	2.59(2.09–3.21)	0.000
3 vs 0	2.60(1.39–4.86)	0.003
Hospitalization in the previous year		
1 vs 0	4.38(3.65–5.24)	0.000
2 vs 0	4.54(2.59–7.96)	0.000
3 vs 0	25.71(8.11–81.53)	0.000
Family member less than 5y		
1 vs 0	1.47(1.23–1.76)	0.000
2 vs 0	9.92(4.14–23.76)	0.000
Family member older than 65y		
1 vs 0	1.37(1.15–1.62)	0.000
2 vs 0	1.82(1.53–2.16)	0.000
Household head’s marriage status		
Married vs not married	1.68(1.10–2.55)	0.016
Divorced vs not married	2.67(1.23–5.78)	0.013
Other vs not married	1.39(0.86–2.27)	0.182
Household head’s education level		
Middle school vs primary school or lower	0.80(0.70–0.92)	0.002
High school vs primary school or lower	0.75(0.61–0.92)	0.006
College or higher vs primary school or lower	0.64(0.42–0.98)	0.038
Household head’s social insurance		
Labor insurance vs none	0.65(0.46–0.90)	0.011
Urban residential insurance vs none	0.89(0.63–1.26)	0.525
NCMS vs none	0.99(0.75–1.32)	0.965
Other social insurance vs none	0.46(0.32–0.67)	0.000

Insig2u 1.469.

Sigma_u 0.480.

rho = 0.065, likelihood-ratio test of rho = 0, P < 0.001.

Random-effects logistic regression, Wald chi2 = 982.64, p < 0.001.

## Discussion

Findings of our study indicate that sociodemographic factors including income and age are important determinants of catastrophic medical expenses, which is consistent with what Shi and colleagues reported in the central and western regions of China [3], as well as with findings in other developing countries [14,16]. Nevertheless, we have two findings that are different from those of other studies [3]. Our first finding is that households with members at age of 5 years or younger had much higher risk of incurring catastrophic medical expenses. There are several explanations. It is quite common in China that parents, even in poor families, are willing to seek the best care possible for their children, especially for their young children who are more susceptible to illness [17]. Moreover, with an exception of Shanghai where an inpatient care insurance program for children under 18 is available, no health insurance programs for children exist in other areas [18].

Our second new finding of the non-linear relationship between income level and incurring catastrophic medical expenses is also not consistent with other studies [3,14,16]. We found that the residents in the poorest income group do not have the highest incidence of catastrophic medical expenses, which may have several explanations. First, this may result from the unmet need of the poorest families that tend to skip or limit their utilization of health services due to financial barriers. The China’s Fourth National Health Services Survey reports that 38% of patients did not seek outpatient care and 14.9% of which were due to financial hardship or high prices of medical services in 2008, and it is also reported that 21% of patients who should be hospitalized were not, 70.3% of which were due to economic difficulties [18]. Therefore, patients’ decisions on health services utilization are strongly affected by their economic capability to pay. The poorest residents often lack adequate financial means to access necessary health services. If people in poverty are unable to seek promptly health services, they can be trapped into a terrible circle of “poverty - > poor health - > poorer. In addition, the incidence of the 2^nd^ income quintile was higher than that of the top income quintile, which is consistent with what Gotsadze and colleagues found in Georgia [14], but is not consistent with results of Shi and colleagues’ study in less developed regions in China [3]. A possible explanation is that the patients with top income levels tend to visit hospitals, especially more expensive hospitals housed by more skillful providers and equipped with more advanced technologies and capacities [5].

Our findings also confirm that hospitalization greatly increases possibilities of incurring catastrophic medical expenses among households. In addition, the odds we estimated could be underestimated if there were residents who should be hospitalized did not go to the hospital. Given the fact that inpatient care is expensive nowadays in China [18], hospitalization is a prominent factor among all factors that result in catastrophic medical expense. The latest national survey revealed that 6.8% of the population gets hospitalized each year. Making the matter worse, uninsured populations are even more susceptible to catastrophic medical expenses [18].

Individual health status is another important determinant of catastrophic medical expenses. Households with family members having chronic diseases tend to suffer catastrophic medical expenses. As the latest national health services survey indicates, persons with chronic illnesses have reached to about 260 million in China [18]. High-risk behavior is one of the contributing factors, but increase in aging populations is another important factor. In addition, relatively speaking, more aging populations reside in eastern China than in central and western China [18,19].

Apart from individual factors, we found that region is also associated with the incidence of catastrophic medical expenses. Although eastern China is the most developed regions in the country, variations in catastrophic medical expenses exist due to economic discrepancies within the regions. People in areas with relatively low income (e.g., rural Weifang) have much higher risk of incurring catastrophic medical expenses than their counterparts in areas with higher income levels (e.g., rural Shanghai and Changzhou). There are several explanations but the most important one is probably related to differences in health insurance programs in different areas. For example, despite of being run by the local government, premiums and reimbursement scales of the New Cooperative Medical Scheme programs differ greatly across areas. With similar rates of enrollment in populations, levels of reimbursement vary in different counties, cities, and provinces, which affect the extent of catastrophic medical expenses.

Urban–rural discrepancies in health expenses persist even in relatively developed areas of China. The incidence of catastrophic medical expenses of rural residents remains much higher than that of urban residents. On the one hand, the rural residents’ income in general is lower than that of the city residents. On the other hand, urban hospitals are better equipped by medical technology than rural hospitals and rural residents are willing to go to the urban hospitals despite of their higher prices [20].

This study had several limitations. First, we surveyed both urban and rural residents in Shanghai and Weifang but did not survey urban residents in Changzhou due to insufficient funding, but our sample size was large, which enabled us to calculate the incidences of catastrophic medical expenses in rural and urban residents separately. Another limitation is that economic data of villages were not available in our datasets although they were used by other studies [3].

We used multi-level model because the data in this study were characteristic of hierarchical structures,and the results verified that correlation did exist within the group. Multi-level model can reflect the micro-level (household level) and macro-level (area-level) independent variables, which ordinary logistic model cannot do. Therefore, as to the hierarchy data, we should use a multi-level model.

Such a large-scale survey with a response rate over 95% could not be completed without the support of the local government. It is similar to the National Health Services Survey (NHSS). In China, the NHSS has been done every five years throughout the China, with response rates above 95% [21], which is also contributed to the support from the central and local government.

## Conclusions

In conclusion, the occurrence of catastrophic medical expenses among Chinese populations is associated with such factors as family with special member, family income, health services utilization, health insurance coverage, family members’ general health status, and regional socioeconomic variations. According to the results, we can adopt some intervention to reduce catastrophic medical expenses.

Firstly, based on the fact that household with members with special age has high risk is a determinant, we can establish targeted health insurance for the special residents such as old residents or very young children.

Secondly, it will be effective way to improve individual health status to reduce use of health services, especially hospitalization according to the result that households with member who hospitalized or suffered chronic diseases were vulnerable to incur catastrophic medical expenses. It is reported that health education can help people form healthy behavior, which will lead to better health status [22,23]. Policy can encourage these health education programs. It is extremely important to take effective intervention measures to ensure patients to receive timely, affordable, and high-quality health care to control and avoid deterioration of their diseases, which will prevent avoidable hospitalizations.

Finally, the result that discrepancies exist in different regions even all the regions are within the developed eastern China shows that it remains challenging according to improving equalization in different regions. Take New Cooperative Medical Scheme for example, it needs to be realized that a mere improvement in coverage is not sufficient, and it is more important to improve the level compensation in the future. The New Cooperative Medical Scheme has achieved the coverage of 90% or higher [18], but the level of compensation varies greatly across areas and is not high in most areas in rural China. Given the current high proportion of out-of-pocket payment [24], a more important step is to increase the level of reimbursement.

## Competing interests

The authors declare that they have no competing interests.

## Authors’ contributions

XL participated in design of study, data analysis and interpretation, statistical methods, manuscript draft. JS participated in statistical methods, interpretation and manuscript draft. JL participated in design and concept of study, interpretation and acquisition of data. YW participated in concept of the study and interpretation. MS participated in interpretation and acquisition of data. CL participated in interpretation and acquisition of data. FC participated in acquisition of data. MH participated in design and concept of study, acquisition of data, acquisition of funding, and supervision. All authors read and approved the final manuscript.

## Pre-publication history

The pre-publication history for this paper can be accessed here:

http://www.biomedcentral.com/1472-6963/13/506/prepub

## Supplementary Material

Additional file 1 Questionnaire related to this article. Click here for file
