# Supplementary material for: Household catastrophic medical expenses in eastern China: determinants and policy implications
Source: BMC Health Serv Res. 2013 Dec 5;13:506. doi: 10.1186/1472-6963-13-506 (PMC4234144; doi:10.1186/1472-6963-13-506)
Supplement: Additional file 1 — Questionnaire related to this article. [file 1472-6963-13-506-S1.doc]

**Questionnaire of Health Status and Health Services**

Household head’s name:

Address：

Telephone：

Date: Investigator

**Table 1 Household’s income and expenses**

| Question(Please fill the blanks in Right column) | Answer |
| --- | --- |
| 1. The number of family members |  |
| 1. The type of household head’s registration：(1) urban (2) rural |  |
| 1. How much did the household spend on daily living in the last year？ |  |
| 1. food expenditure |  |
| 1. clothing and daily necessities expenditure |  |
| 1. traffic and communications expenditure |  |
| 1. housing, water, electricity and fuel expenditure |  |
| 1. culture, education and entertainment expenditure |  |
| 1. medicines, medical services and supplies expenditure |  |
| 1. other |  |
| 1. How much was the household’s income in the last year? |  |

**Table 2 Individual health status and hospitalization**

| Code(01: household head, please code other members from the oldest to the youngest) | 01 | 02 | 03 | 04 | 05 | 06 | 07 |
| --- | --- | --- | --- | --- | --- | --- | --- |
| 1. Age |  |  |  |  |  |  |  |
| 1. Sex：(1)male (2)female |  |  |  |  |  |  |  |
| 1. Ethnicity：(1)han (2) other |  |  |  |  |  |  |  |
| 1. Marriage status (applied for older than 15 y): 2. unmarried 3. married 4. divorced 5. other |  |  |  |  |  |  |  |
| 1. Education (applied for older than 15 y): 2. not finish elementary school 3. never went school but know some words 4. elementary school 5. middle school 6. high school 7. vocational high school 8. college 9. undergraduate and above |  |  |  |  |  |  |  |
| 1. Occupation (applied for older than 15 y)：   manager in government and institutions   1. manager in enterprises 2. private entrepreneurs and managers 3. professional and technical personnel 4. ordinary administrative in government, enterprises and institutions 5. primary businesses 6. employees in business or service industry 7. urban worker 8. farmers engaged in non-agricultural labor in urban area 9. farmers engaged in non-agricultural labor in urban area 10. farmer 11. student 12. tired 13. unemployed |  |  |  |  |  |  |  |
| 1. Do you have any social health insurance? 2. none 3. labor insurance 4. urban residents insurance 5. New Cooperative Medical Scheme 6. other |  |  |  |  |  |  |  |
| 1. Do you have private health insurance: (1)yes (2)no |  |  |  |  |  |  |  |
| 1. Have you got chronic diseases? (1)yes (2)no |  |  |  |  |  |  |  |
| 1. Have you been hospitalized in the last year? If yes, how many times? |  |  |  |  |  |  |  |
